# Hippocampal disruptions of synaptic and astrocyte metabolism are primary events of early amyloid pathology in the 5xFAD mouse model of Alzheimer’s disease

**DOI:** 10.1038/s41419-021-04237-y

**Published:** 2021-10-16

**Authors:** Jens V. Andersen, Niels H. Skotte, Sofie K. Christensen, Filip S. Polli, Mohammad Shabani, Kia H. Markussen, Henriette Haukedal, Emil W. Westi, Marta Diaz-delCastillo, Ramon C. Sun, Kristi A. Kohlmeier, Arne Schousboe, Matthew S. Gentry, Heikki Tanila, Kristine K. Freude, Blanca I. Aldana, Matthias Mann, Helle S. Waagepetersen

**Affiliations:** 1 Department of Drug Design and Pharmacology, Faculty of Health and Medical Sciences, University of Copenhagen, Copenhagen, Denmark; 2 Novo Nordisk Foundation Center for Protein Research, Faculty of Health and Medical Sciences, University of Copenhagen, Copenhagen, Denmark; 3 Department of Neuroscience, Dorris Neuroscience Center, The Scripps Research Institute, La Jolla, CA USA; 4 Neuroscience Research Center, Neuropharmacology Institute, Kerman University of Medical Sciences, Kerman, Iran; 5 Department of Molecular and Cellular Biochemistry, College of Medicine, University of Kentucky, Lexington, KY USA; 6 Department of Veterinary and Animal Sciences, Faculty of Health and Medical Sciences, University of Copenhagen, Copenhagen, Denmark; 7 Markey Cancer Center, Lexington, KY USA; 8 Department of Neuroscience, College of Medicine, University of Kentucky, Lexington, KY USA; 9 A. I. Virtanen Institute for Molecular Sciences, University of Eastern Finland, Kuopio, Finland

**Keywords:** Proteomics, Alzheimer's disease, Molecular neuroscience, Alzheimer's disease

## Abstract

Alzheimer’s disease (AD) is an unremitting neurodegenerative disorder characterized by cerebral amyloid-β (Aβ) accumulation and gradual decline in cognitive function. Changes in brain energy metabolism arise in the preclinical phase of AD, suggesting an important metabolic component of early AD pathology. Neurons and astrocytes function in close metabolic collaboration, which is essential for the recycling of neurotransmitters in the synapse. However, this crucial metabolic interplay during the early stages of AD development has not been sufficiently investigated. Here, we provide an integrative analysis of cellular metabolism during the early stages of Aβ accumulation in the cerebral cortex and hippocampus of the 5xFAD mouse model of AD. Our electrophysiological examination revealed an increase in spontaneous excitatory signaling in the 5xFAD hippocampus. This hyperactive neuronal phenotype coincided with decreased hippocampal tricarboxylic acid (TCA) cycle metabolism mapped by stable ^13^C isotope tracing. Particularly, reduced astrocyte TCA cycle activity and decreased glutamine synthesis led to hampered neuronal GABA synthesis in the 5xFAD hippocampus. In contrast, the cerebral cortex of 5xFAD mice displayed an elevated capacity for oxidative glucose metabolism, which may suggest a metabolic compensation in this brain region. We found limited changes when we explored the brain proteome and metabolome of the 5xFAD mice, supporting that the functional metabolic disturbances between neurons and astrocytes are early primary events in AD pathology. In addition, synaptic mitochondrial and glycolytic function was selectively impaired in the 5xFAD hippocampus, whereas non-synaptic mitochondrial function was maintained. These findings were supported by ultrastructural analyses demonstrating disruptions in mitochondrial morphology, particularly in the 5xFAD hippocampus. Collectively, our study reveals complex regional and cell-specific metabolic adaptations in the early stages of amyloid pathology, which may be fundamental for the progressing synaptic dysfunctions in AD.

## Introduction

Alzheimer’s disease (AD) is a complex neurodegenerative disorder characterized by cerebral accumulation of amyloid-β (Aβ) plaques and neurofibrillary tangles [1, 2]. Aβ deposition leads to dysfunctional synaptic signaling and neuronal death, progressing over decades before clinical symptoms of dementia arise [2, 3]. This long preclinical phase of AD has been described as the window of potential disease intervention [2, 4]. Hence, understanding the early mechanisms behind AD pathology is imperative in finding a treatment. A progressive decrease in brain glucose metabolism is a robust marker of AD development and is correlated with dementia symptoms [5]. However, differential changes in regional brain energy metabolism already emerge in the preclinical phase of AD, suggesting an important metabolic component of early AD pathology [6, 7].

Cerebral accumulation of Aβ initiates a complex cascade of cellular responses [8]. Particularly astrocytes, a highly abundant glial cell type in the brain, undergo extensive changes upon Aβ exposure [9–11]. Neurons and astrocytes collaborate in a tightly coupled metabolic network [12]. Astrocytes are crucial for neurotransmitter recycling as they provide neurons with glutamine, an essential substrate for replenishment of the neurotransmitter glutamate and GABA pools [13, 14]. Neurotransmitter recycling is closely linked to cellular energy metabolism as the tricarboxylic acid (TCA) cycle, located within the mitochondria, provides α-ketoglutarate for glutamate synthesis, being the precursor of both GABA and glutamine synthesis [14, 15]. Mounting evidence suggests that changes in mitochondrial function and cellular metabolism are implicated in the early stages of AD [16], making it crucial to explore and understand how these changes relate to the metabolic interplay of neurons and astrocytes.

The aim of this study was to provide an integrative investigation on regional cellular metabolism during the early developing stages of cerebral amyloid pathology in the 5xFAD mouse model of AD [17]. 5xFAD mice develop intraneuronal Aβ accumulation at 1.5 months (mo) of age, with extracellular amyloid plaques at 2 mo [17] and impaired working memory at 4–6 mo [17, 18]. For this study, 5xFAD mice were used at 2 and 4 mo of age, representing two early stages of amyloid pathology. By applying a wide array of experimental approaches, including proteomics, metabolomics, electrophysiology, ^13^C isotope tracing, functional mitochondrial analyses and electron microscopy, we demonstrate complex regional and cell-specific dysfunctions of metabolism and signaling in the 5xFAD brain during the early stages of amyloid pathology. Our results suggest that early regional metabolic alterations, of both neurons and astrocytes, may be fundamental for the progressive dysfunctions in AD.

## Results

### Perturbations of the brain proteome and metabolome are limited during early amyloid pathology

**Fig. 1:**
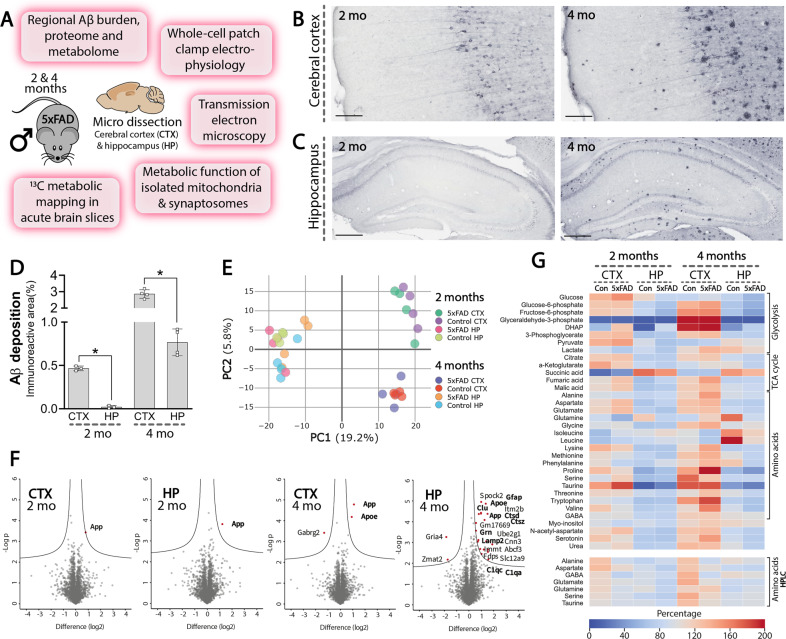
Hippocampal and cerebral cortical amyloid-β (Aβ) burden, proteome and metabolome of 5xFAD mice. **A** Experimental overview of the study: male 5xFAD and control mice were utilized at 2 and 4 months (mo) of age. Representative Aβ staining of the cerebral cortex (**B**) and the hippocampus (**C**) of 2 and 4 mo old 5xFAD mice. Scale bars: **B**: 100 µm, **C**: 250 µm. **D** Quantification of regional Aβ immunoreactive areas in the cerebral cortex and hippocampus of the 5xFAD mice, mean ± SEM, *n* = 3-4, Student’s paired *t*-test. **E** Principal component analysis (PCA) plot of the 5xFAD proteome shows distinct clustering of samples based on brain region and age, *n* = 4. **F** Volcano plots displaying significant alterations in protein expression in the 5xFAD hippocampus and cerebral cortex. Proteins in the upper left quadrant signify decreased expression, whereas protein in the upper right quadrant signify increased expression. Statistical analysis of the proteome and metabolome is described in detail in the ‘Materials and methods’ section. **G** Heatmap displaying normalized metabolite abundance of glycolytic and TCA cycle intermediates, amino acids and other metabolites in microwave fixated brain tissue from 5xFAD mice, *n* = 6–7, Student’s paired *t*-test. Blue tones denote reduced abundance, whereas red tones denote elevated abundance. CTX: cerebral cortex, HP: hippocampus.

To elucidate how early amyloid pathology affects regional brain metabolism in the 5xFAD mouse we combined several experimental approaches (Fig. 1A). First, we assessed the regional Aβ burden of the 5xFAD mice in the two primary brain regions affected in AD: the cerebral cortex (CTX, Fig. 1B) and hippocampus (HP, Fig. 1C). At 2 mo, intraneuronal Aβ accumulation was observed in the cerebral cortex, staining both soma and dendrites of pyramidal neurons in deep cortical layers. In contrast, limited Aβ accumulation was found in the hippocampus at 2 mo. At 4 mo, the 5xFAD cerebral cortex displayed a more intense intraneuronal Aβ staining as well as scattered Aβ plaques, which were also found in the hippocampus. We found no Aβ immunoreactivity in the brain of wild-type control mice (Fig. S1). Quantification of Aβ immunoreactive areas revealed a significantly larger amyloid burden in the 5xFAD cerebral cortex than in the hippocampus at both 2 and 4 mo (Fig. 1D) and confirmed that both ages correspond to early stages of amyloid pathology in the 5xFAD mice [17, 18].

Next, to get an unbiased overview of how early amyloid pathology may impact regional systems biology, we mapped the 5xFAD cerebral cortical and hippocampal proteome by mass spectrometry (MS). In total, we identified 5729 proteins and found a strong reproducibility (Pearson correlation: 0.93–0.97) between biological samples (Fig. S2A, B). Principal component analysis (PCA) revealed a clear sample stratification based on brain region and age (Fig. 1E). Across the eight groups we found 1997 significant protein differences (FDR < 0.05) (Fig. S2C), predominantly related to the difference between brain regions and age, which is comparable to our previous proteomic study of Huntington’s disease [19]. When comparing protein changes between control and 5xFAD mice, we found that amyloid precursor protein (APP), the precursor protein of Aβ, was significantly increased in both the 5xFAD cerebral cortex and hippocampus at both 2 and 4 mo (Fig. 1F). Furthermore, at 4 mo we observed elevated expression of ApoE in both brain regions, and of GFAP in the hippocampus. Furthermore, we identified 16 additional proteins with increased expression in the 5xFAD hippocampus at 4 mo, primarily related to the complement system (Clu, C1qa, C1qc), lysosomal function, and protein degradation (Grn, Lamp2, Ctsz, Ctsd). To supplement the proteome, we determined the metabolite abundance by MS analysis in microwave fixated brains of 5xFAD and control mice. As observed for the proteome, the metabolome clustered according to brain region and age (Fig. 1G). However, the abundance of all measured glycolytic intermediates, TCA cycle intermediates and amino acids, was unchanged between 5xFAD and control mice, which was further confirmed by high-performance liquid chromatography (HPLC) analysis (Fig. 1G and absolute amino acid amounts in Tables S1 and S2). Collectively, the overall maintained proteome and metabolome suggests that abnormalities in protein expression and metabolite abundance are not initiating pathological processes during early amyloid pathology of the 5xFAD brain.

### Early hippocampal disruptions of spontaneous excitatory signaling

**Fig. 2:**
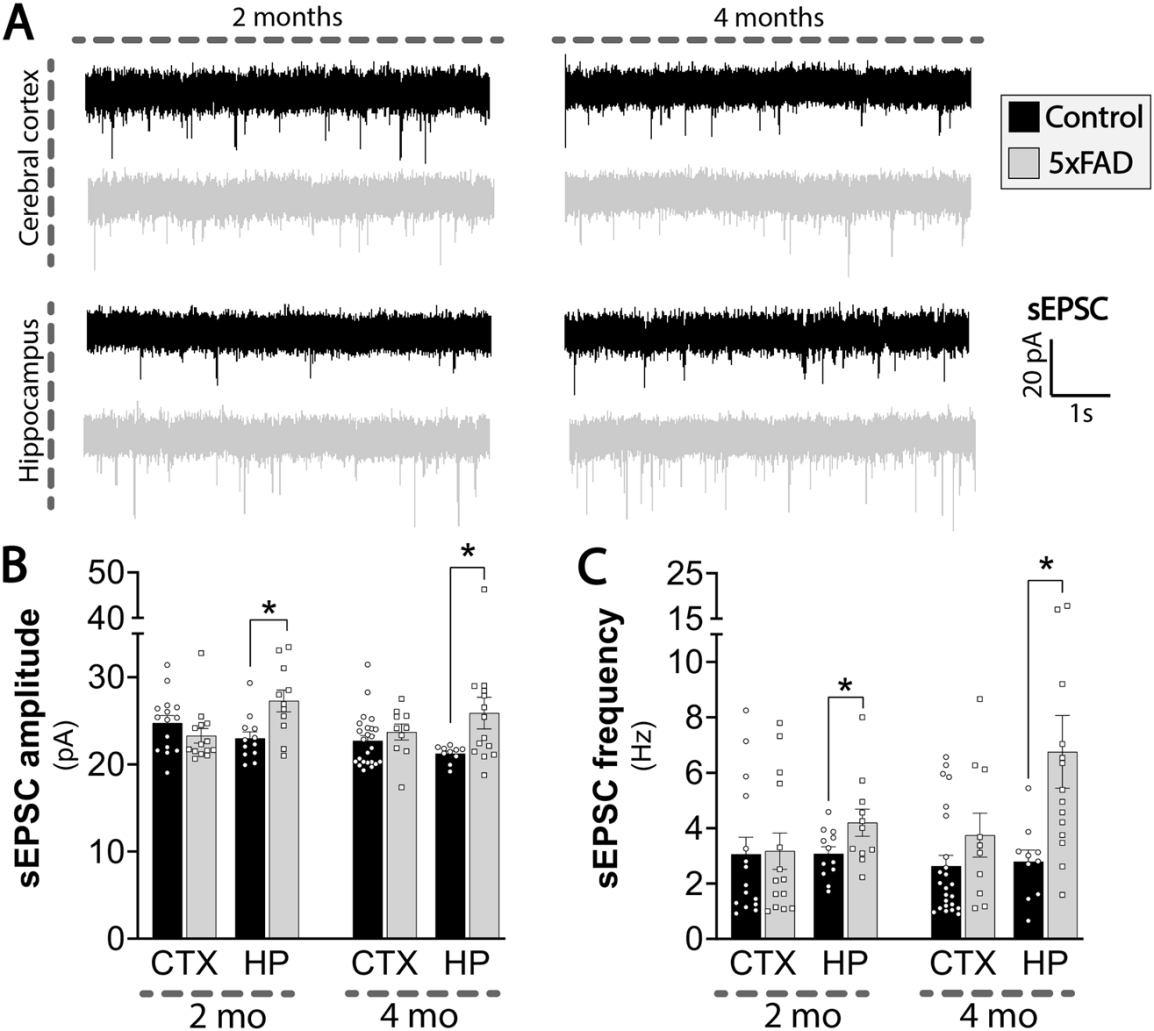
Elevated spontaneous excitatory synaptic activity in 5xFAD hippocampal neurons. Neuronal signaling was assessed by whole-cell patch-clamp electrophysiology in brain slices of 2 and 4 months (mo) old 5xFAD and control mice. **A** Representative recordings of spontaneous excitatory post-synaptic currents (sEPSCs) (black traces: control, gray traces: 5xFAD). Quantification of amplitude (**B**) and frequency (**C**) in cerebral cortical and hippocampal slices of 2 and 4 mo old 5xFAD and control mice. sEPSCs were obtained in voltage-clamp, gap-free mode, with a voltage command set to −60 mV. CTX: cerebral cortex, HP: hippocampus. Mean ± SEM, *n* = 10–24 from 4–6 pairs of animals, Student’s unpaired *t*-test or Mann-Whitney test.

As dysfunctional synaptic signaling is a hallmark of AD [20], we next investigated functional neuronal signaling in acutely isolated brain slices by whole-cell patch-clamp recordings of pyramidal neurons in the cerebral cortex and the CA1 region of the hippocampus. All measured passive electrical membrane properties were unchanged, in both the cerebral cortex and hippocampus of 5xFAD mice at both 2 and 4 mo (Tables S3 and S4). However, a clear increase in spontaneous excitatory synaptic activity was found in the 5xFAD hippocampus (Fig. 2A). Both the amplitude (Fig. 2B) and frequency (Fig. 2C) of the spontaneous excitatory post-synaptic currents (sEPSCs) were significantly higher in 5xFAD hippocampus at both 2 and 4 mo. In contrast, no changes in sEPSCs were found in the cerebral cortex (Fig. 2A–C). These results demonstrate early dysfunctions of neuronal signaling selectively in the 5xFAD hippocampus, suggesting functional changes in excitatory signaling.

### Hampered functional energy metabolism and neurotransmitter recycling

**Fig. 3:**
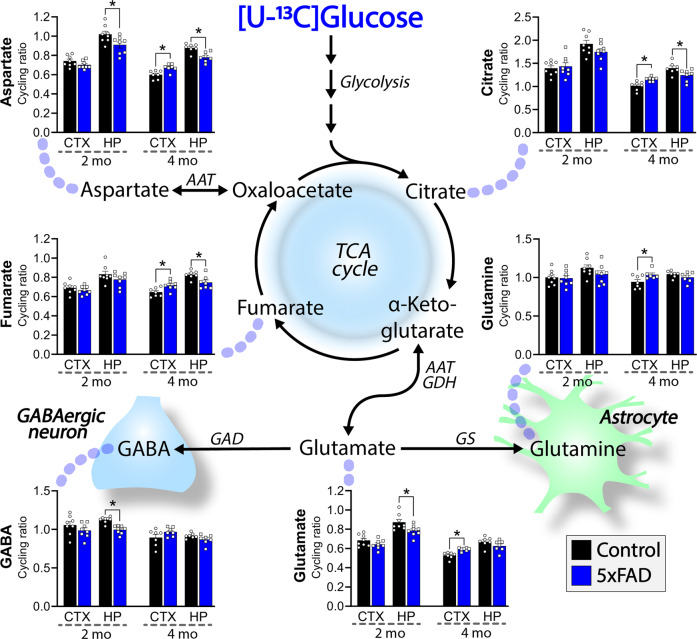
Disrupted glucose metabolism in cerebral cortical and hippocampal brain slices of 5xFAD mice. Cycling ratios, reflecting the rate of the TCA cycle, calculated from metabolism of [U-^13^C]glucose in acutely isolated cerebral cortical and hippocampal brain slices of 2 and 4 months (mo) old 5xFAD mice. GABA and glutamine are selectively synthesized in neurons and astrocytes, respectively. AAT: aspartate aminotransferase, CTX: cerebral cortex, GAD: glutamate decarboxylase, GDH: glutamate dehydrogenase, GS: glutamine synthetase, HP: hippocampus, PAG: phosphate-activated glutaminase. Mean ± SEM, *n* = 6–8, Student’s unpaired *t*-test with Benjamini-Hochberg correction.

**Fig. 4:**
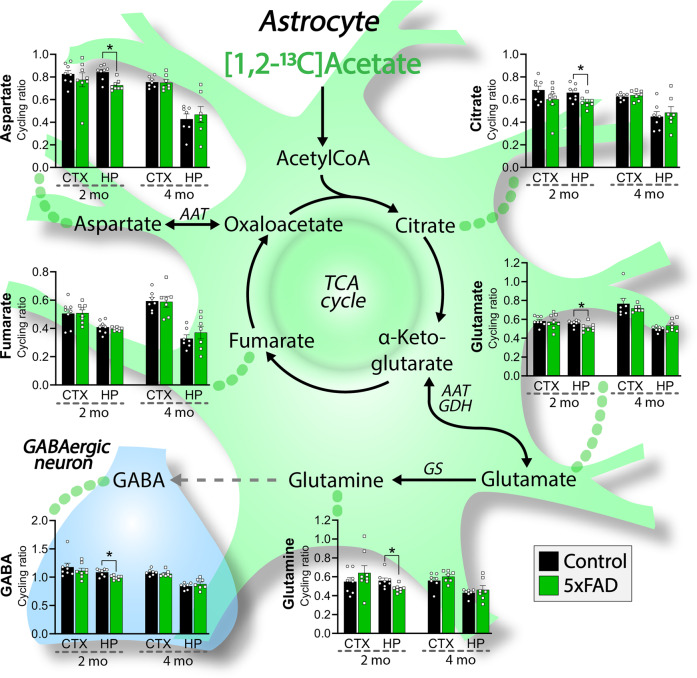
Hampered astrocyte metabolism and glutamine synthesis in 5xFAD hippocampal brain slices. Cycling ratios, reflecting the rate of the TCA cycle, calculated from metabolism of [1,2-^13^C]acetate in acutely isolated cerebral cortical and hippocampal brain slices of 2 and 4 months (mo) old 5xFAD mice. [1,2-^13^C]Acetate is predominantly metabolized in astrocytes. Astrocytes synthesize glutamine, which is essential for the replenishment of the neuronal GABA pools. AAT: aspartate aminotransferase, CTX: cerebral cortex, GAD: glutamate decarboxylase, GDH: glutamate dehydrogenase, GS: glutamine synthetase, HP: hippocampus, PAG: phosphate-activated glutaminase. Mean ± SEM, *n* = 7–8, Student’s unpaired *t*-test with Benjamini-Hochberg correction.

Given that neurotransmission is tightly coupled to cellular metabolism [21], we next investigated if the observed changes in neuronal signaling could be linked to functional metabolic alterations. To this end, we incubated acutely isolated cerebral cortical and hippocampal slices in the presence of ^13^C-enriched energy substrates to functionally map cellular energy metabolism (Figs. 3 and 4). First, we provided the slices with [U-^13^C]glucose, which is metabolized through glycolysis to pyruvate and gives rise to ^13^C labeling in lactate and alanine, both of which were unchanged (Fig. S3), indicating sustained glycolytic activity in the 5xFAD brain slices. Further oxidative metabolism of [U-^13^C]glucose leads to ^13^C accumulation in TCA cycle intermediates and connected amino acids, from which the cycling ratio, reflecting the rate of TCA cycle, was calculated [22]. In the hippocampal slices of 2 mo old 5xFAD mice, we found reduced cycling ratios from the metabolism of [U-^13^C]glucose, in aspartate, glutamate, and GABA, whereas no changes were observed in the cerebral cortical slices at this age (Fig. 3). In 4 mo old 5xFAD mice, reduced hippocampal metabolism of [U-^13^C]glucose was again observed, as decreased cycling ratios in aspartate, citrate, and fumarate, signifying reduced TCA cycle activity in this region. Intriguingly, at 4 mo, a prominent increase in the TCA cycle activity was found in cerebral cortical slices of 5xFAD mice, as elevated cycling ratios were observed in aspartate, citrate, fumarate, glutamate, and glutamine from the metabolism of [U-^13^C]glucose. This differential regional metabolic phenotype in the 5xFAD brain was further supported by the metabolism of an alternative substrate, the ketone body [U-^13^C]β-hydroxybutyrate (Fig. S4). Next, we provided the slices with [1,2-^13^C]acetate, a substrate which primarily enters astrocytic energy metabolism [23] (Fig. 4). In hippocampal slices of 2 mo old 5xFAD mice, we found reduced cycling ratios from the metabolism of [1,2-^13^C]acetate, in citrate, aspartate, glutamate, glutamine, and GABA, which indicates hampered astrocyte TCA cycle activity. The ^13^C enrichment in GABA from the metabolism of [1,2-^13^C]acetate in the brain slices results from the functional transfer of glutamine from astrocytes to neurons [23, 24]. The results therefore suggest that diminished astrocyte glutamine support hampers neuronal GABA synthesis during early amyloid pathology in the 5xFAD hippocampus. Interestingly, [1,2-^13^C]acetate metabolism was found to be unchanged in the cerebral cortical slices from 2 mo old 5xFAD mice and in both regions at 4 mo, signifying that the early changes in hippocampal astrocyte metabolism are transient. In addition, we found a maintained capacity for [U-^13^C]glutamate uptake and metabolism in the 5xFAD slices (Fig. S5). Taken together, the results above demonstrate region-specific shifts in functional brain metabolism in the 5xFAD mouse. Particularly, the hippocampus was affected as reduced glucose metabolism was found at both 2 and 4 mo, whereas the increase in cerebral cortical glucose metabolism at 4 mo could signify a metabolic compensation in the 5xFAD brain.

### Maintained non-synaptic mitochondrial function

**Fig. 5:**
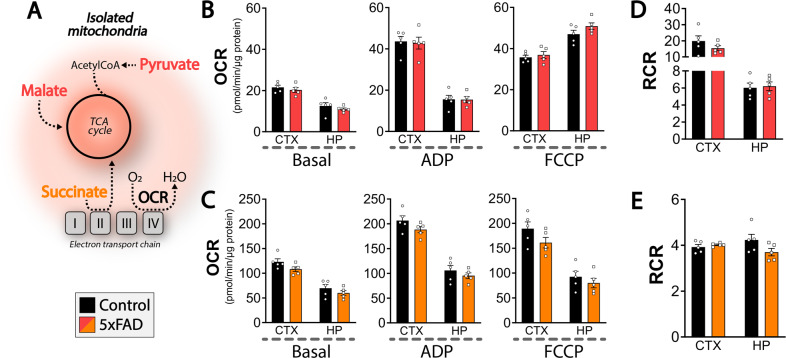
Non-synaptic mitochondrial function is maintained in the 5xFAD brain. Analysis of oxygen consumption rate (OCR) of isolated non-synaptic cortical and hippocampal mitochondria of 4 months old 5xFAD mice. **A** The mitochondria were provided with two different substrate combinations: pyruvate in combination with malate (**B**, **D**) or succinate (**C**, **E**). The mitochondria were stimulated with ADP, inducing coupled respiration and the mitochondrial uncoupler FCCP leading to maximal uncoupled respiration. Basal refers to non-stimulated OCR. The respiratory control ratio (RCR) is a general measure of mitochondrial function. CTX: cerebral cortex, HP: hippocampus. Mean ± SEM, *n* = 5, Student’s unpaired *t*-test with Benjamini-Hochberg correction.

The metabolic phenotype of reduced hippocampal but elevated cerebral cortical glucose metabolism observed in 4 mo old 5xFAD mice may indicate central perturbations of cellular metabolism. We therefore went on to investigate mitochondrial function by measuring the oxygen consumption rate (OCR) of acutely isolated non-synaptic mitochondria from 4 mo old control and 5xFAD mice (Fig. 5). The isolated mitochondria were challenged by sequential addition of ADP, stimulating coupled respiration, and FCCP to induce maximal uncoupled respiration (Fig. 5A). In the presence of pyruvate and malate as respiratory substrates, no changes were observed in basal, ADP-, or FCCP-stimulated OCR of non-synaptic mitochondria between 5xFAD and control mice (Fig. 5B). Likewise, when provided with succinate, similar OCRs were observed between mitochondria of control and 5xFAD mice in both regions (Fig. 5C). The respiratory control rate (RCR), a measure of overall mitochondrial function [25], was also found to be similar for both the malate/pyruvate (Fig. 5D) and succinate (Fig. 5E) conditions. These results demonstrate maintained non-synaptic mitochondrial function in the 5xFAD brain.

### Diminished energetics of the hippocampal synapse

**Fig. 6:**
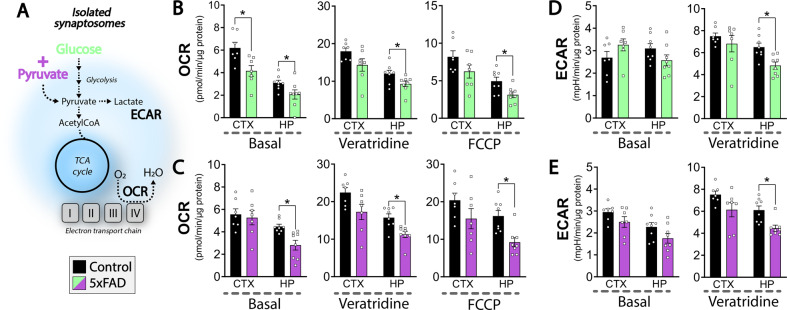
Impaired oxidative and glycolytic capacity of hippocampal synaptosomes of 5xFAD mice. Analysis of oxygen consumption rate (OCR) and extracellular acidification rate (ECAR) of isolated cortical and hippocampal synaptosomes of 4 months old 5xFAD mice. **A** The synaptosomes were provided with two different substrate combinations: glucose (**B**, **D**) or glucose in combination with pyruvate (**C**, **E**). The synaptosomes were stimulated with veratridine, a neurotoxin inhibiting closure of voltage-gated sodium channels leading to depolarization, and with the mitochondrial uncoupler FCCP inducing maximal uncoupled respiration. Basal refers to non-stimulated OCR and ECAR. CTX: cerebral cortex, HP: hippocampus. Mean ± SEM, *n* = 7–8, Student’s unpaired *t*-test with Benjamini-Hochberg correction.

Since synaptic dysfunction is a hallmark of AD development [3], we next investigated the metabolic function of isolated synaptosomes from 4 mo old control and 5xFAD mice (Fig. 6). The synaptosomes were challenged by the addition of depolarizing neurotoxin veratridine and by FCCP (Fig. 6A). With glucose as the only respiratory substrate, synaptosomes of both the 5xFAD cerebral cortex and hippocampus displayed reduced basal OCR (Fig. 6B). When challenged with veratridine and FCCP, the OCRs of the hippocampal synaptosomes of 5xFAD mice were found to be decreased compared to controls (Fig. 6B). When pyruvate was added to enhance the metabolic capacity of the synaptosomes, the basal OCR of the 5xFAD hippocampal synaptosomes was significantly decreased, whereas this was not the case for synaptosomes of the cerebral cortex (Fig. 6C). Furthermore, both the depolarized and uncoupled OCRs of the hippocampal synaptosomes were again reduced when pyruvate was present (Fig. 6C). Since synaptosomes are membrane-enclosed structures with a functional cytosol, the extracellular acidification rate (ECAR) can be determined as a measure of glycolytic activity. No changes in basal ECAR between 5xFAD and control mice were observed in the presence of glucose (Fig. 6D). However, when the synaptosomes were depolarized, a significant decrease in ECAR was observed in the hippocampal synaptosomes of the 5xFAD mice (Fig. 6D), which was also observed when pyruvate was present as an additional substrate (Fig. 6E). These results signify that the basal glycolytic function is maintained in the 5xFAD hippocampal synaptosomes, but may become insufficient during depolarization. The deficient hippocampal mitochondrial and glycolytic capacity was also observed when the synaptosomes were supplemented with the ketone body β-hydroxybutyrate (Fig. S6). Taken together, these results demonstrate extensive dysfunctions of both mitochondrial and glycolytic function in the synapses of the 5xFAD hippocampus.

### Ultrastructural synaptic impairments and mitochondrial dysfunction

**Fig. 7:**
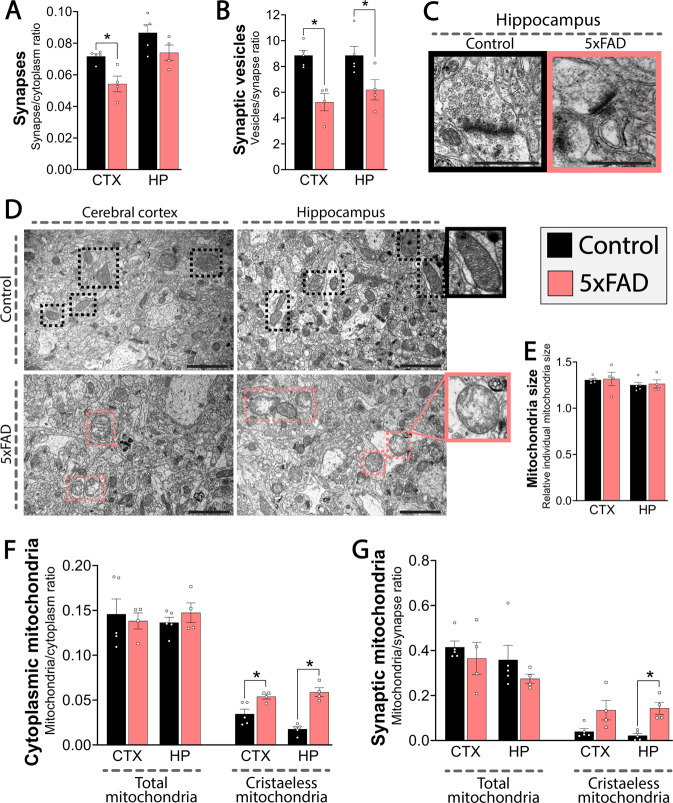
Synaptic and mitochondrial changes in the 5xFAD brain. Ultrastructural analyses by transmission electron microscopy (TEM) evaluating mitochondria, synapses, and synaptic vesicles in fixated cerebral cortical and hippocampal tissues of 4 months old 5xFAD mice. **A** Number of synapses relative to cytoplasm. **B** Number of vesicles per synapse. **C** Representative images of hippocampal synapses of control and 5xFAD. Scale bars: 2 µm. **D** Representative images of mitochondrial abundance and structure in cerebral cortex and hippocampus of control and 5xFAD mice. Scale bars: 2 µm. **E** Relative mitochondrial size. **F**, **G** Analysis of cytosolic (**F**) and synaptic (**G**) mitochondria. Both number of total and number of cristaeless mitochondria were assessed relative to cytoplasm (**F**) or synapses (**G**). CTX: cerebral cortex, HP: hippocampus. Mean ± SEM, *n* = 4–5, Student’s unpaired *t*-test with Benjamini-Hochberg correction.

To investigate if the observed functional alterations of synaptic signaling and metabolism in the 5xFAD brain had morphological correlates, we finally performed ultrastructural analyses on fixated brain tissue of 4 mo old control and 5xFAD mice by transmission electron microscopy (TEM) (Fig. 7). First, we assessed the number of synapses, which was significantly reduced in the 5xFAD cerebral cortex (Fig. 7A). In addition, we found that the number of synaptic neurotransmitter vesicles was reduced in both the cerebral cortex and hippocampus (Fig. 7B, C). To substantiate the functional mitochondrial deficits, we investigated mitochondrial morphology in the 5xFAD brains (Fig. 7D). There was no difference in the overall mitochondrial size between control and 5xFAD mice (Fig. 7E). In the cytoplasm and in the synapse, the total number of mitochondria was likewise unchanged (Fig. 7F, G). However, we observed a significant increase in the occurrence of cristaeless mitochondria in the cytoplasm of both the cerebral cortex and hippocampus of the 5xFAD mice (Fig. 7F) and in the 5xFAD hippocampal synapses (Fig. 7G). Since the cristae house the machinery of mitochondrial energy production, the ultrastructural analyses support the observations of metabolic decline, and furthermore indicate that the alterations in mitochondrial function may drive the bioenergetics deficits in early AD.

## Discussion

Our integrative investigation of regional brain metabolism in the 5xFAD mouse demonstrates regional and cell-specific metabolic impairments during the early stages of amyloid pathology. In the 5xFAD hippocampus, we found dysfunctional excitatory neuronal signaling, hampered cellular metabolism, and alterations in the proteome. In contrast, the 5xFAD cerebral cortex displayed a generally maintained and even elevated metabolic capacity. Synapses and mitochondria were affected at the ultrastructural level in both the cerebral cortex and hippocampus of the 5xFAD mice, but this was only functionally reflected in the hippocampus, suggesting particularly strong initial metabolic impacts of early amyloid pathology in this region.

### Regional changes in brain metabolism in AD

The metabolic hallmark of AD is an overall decrease in brain glucose utilization [2, 5, 7], which was also observed in the 5xFAD hippocampal slices even before significant Aβ plaque formation. Interestingly, several studies have reported elevated metabolic rates in cortical regions of AD patients prior to detectable amyloid accumulation [6, 7, 26, 27]. This is in line with our findings of enhanced neuronal metabolism of [U-^13^C]glucose in the cerebral cortex of 4 mo old 5xFAD mice. Such elevated glucose metabolism has not been reported for the hippocampus and could indicate that the cerebral cortex is able to metabolically compensate during early AD development. Interestingly, neuronal network activity has been associated with Aβ accumulation, as the highly active default mode network (DMN) of the brain [28] is particularly susceptible towards Aβ accumulation [29, 30]. In addition, it was recently shown that distal Aβ aggregation in the DMN can cause metabolic changes in adjacent connected brain regions [31], which may, in part, explain our observations of hippocampal metabolic decline prior to detectable amyloid accumulation in the 5xFAD mice. Great effort is currently going into large-scale omics studies of the AD brain [32–34]. Our mapping of the regional 5xFAD proteome revealed few changes in the early phases of amyloid pathology. However, several of our findings are in accordance with previous reports, including elevated GFAP, APP, Clusterin (Clu), and Cathepsin-D (Ctsd) expression in the 5xFAD hippocampus [35, 36]. Our finding of elevated hippocampal expression of proteins related to the complement system and lysosomal function has also been observed in human AD brain samples [37, 38]. Notably, we also found elevated expression of the initiating factors of the complement system C1qa and C1qc in the 5xFAD hippocampus. C1q is able to mediate synapse elimination [39, 40] and may aid in explaining the observed aberrant signaling synaptic function in this region. This is further in line with the ultrastructural analysis showing reduced number of synapses and neurotransmitter vesicles in the 5xFAD mice. However, since the 5xFAD cerebral cortical proteome was largely maintained, the synaptic loss in this region is likely mediated by other mechanisms. Substantial changes of the brain proteome have been described in older 5xFAD mice (8–12 mo) [33, 41], which may suggest that the few differentially expressed proteins found in this study could be initiating larger remodulations of the proteome. Our observation of a maintained cerebral metabolome has also been reported for older 5xFAD mice (6 mo) [42] and underlines the importance of applying functional metabolic studies when investigating the metabolic impacts of early AD pathology.

### Astrocytes and neurotransmitter recycling in AD

Astrocytes display complex pathological profiles in AD and can become either reactive with a hypertrophic phenotype or display cellular atrophy with loss of function [9–11, 43]. It has been hypothesized that astrocytes may lose homeostatic functions and provide less metabolic support for neurons in AD [8, 44, 45]. Here we found evidence of altered hippocampal astrocyte metabolism in 5xFAD mice already at 2 mo of age. Functional transfer of glutamine, from astrocytes to neurons, is maintained in our slice incubation set-up [24], indicating that the decreased ^13^C labeling in GABA from the metabolism of [1,2-^13^C]acetate is caused by insufficient provision of astrocyte glutamine. Expression of glutamine synthetase (GS), the astrocyte-specific enzyme converting glutamate into glutamine, has been found to be reduced in both human samples and mouse models of AD [46, 47]. We recently demonstrated that hampered astrocyte glutamine supply impaired neuronal GABA synthesis in 8 mo old 5xFAD mice [48]. However, it is striking that this astrocytic deficit was present even before detectable amyloid accumulation in the 5xFAD hippocampus, suggesting a fundamental role of dysfunctional astrocyte metabolism in early AD pathology. Our electrophysiological experiments further revealed a hyperactive neuronal phenotype selectively in the 5xFAD hippocampus. These findings are supported by reports demonstrating elevated neuronal activity in other AD models [49–51] and extend this neuronal phenotype to the 5xFAD mouse. It is intriguing that the 5xFAD hippocampal hyperactivity coincided with reduced astrocyte glutamine transfer and decreased neuronal GABA synthesis. Reduced GABA synthesis could disrupt the neuronal inhibitory tone and hereby contribute to the hyperexcitable neuronal phenotype. Furthermore, since glutamate uptake is a highly energy requiring process [14, 52], reductions of astrocyte TCA cycle activity could impair glutamate uptake capacity. Interestingly, it was recently demonstrated that dysfunctional astrocyte glutamate clearance indeed could drive the early hippocampal hyperexcitability [53]. However, when we explored this, we found no evidence of impaired astrocyte uptake and metabolism of [U-^13^C]glutamate (Fig. S5). However, our experimental metabolic set-up with prolonged incubation times may not reveal subtle impairments of cellular glutamate uptake and it would be valuable to further investigate the metabolic link between astrocyte energetics and glutamate uptake in early AD. Interestingly, the observed changes in astrocyte energy and neurotransmitter homeostasis were transient, as no differences in [1,2-^13^C]acetate metabolism were found in hippocampal slices from 5xFAD mice at 4 mo of age. Dysfunctional astrocyte metabolism, beyond deficient glutamine synthesis, has been reported in several AD model systems [54–56]. However, the fact that regional astrocyte metabolism is transiently affected underlines the complexity of metabolic adaptations in the early stages of amyloid pathology [57] and further studies are needed to elucidate the underlying mechanisms.

### Mitochondrial and synaptic metabolic function in AD

Mitochondrial dysfunction is recognized as an early pathological event in AD, manifesting as alterations in mitochondrial morphology, production of reactive oxygen species and oxidative phosphorylation [16, 58, 59]. An important, but often overlooked, aspect of brain mitochondrial function is the region of origin. Individual brain regions display distinct differences in metabolic capacity making it crucial to investigate mitochondrial functionality with respect to the specific brain regions [60, 61]. Here, we found maintained non-synaptic mitochondrial oxygen consumption in both the hippocampus and cerebral cortex of 4 mo old 5xFAD mice. In contrast, we found prominent impairments of mitochondrial function in the 5xFAD hippocampal synaptosomes, which may be attributed to pronounced synaptic mitochondrial Aβ accumulation of the 5xFAD model [62]. However, in the light of the much larger amyloid burden in the cerebral cortex compared to the hippocampus, Aβ alone is not a satisfactory explanation of the regional synaptic dysfunction. When the 5xFAD synaptosomes were provided with glucose as the only substrate, we observed a decreased basal OCR in both the cerebral cortex and hippocampus, which is supported by impaired glycolytic function in the AD brain [63]. Indeed, we found reduced glycolytic capacity (ECAR) in the 5xFAD hippocampal synaptosomes when metabolically challenged by depolarization. In an attempt to enhance the metabolic capacity by circumventing glycolysis, we provided the synaptosomes with either pyruvate (Fig. 6C, E) or the ketone body β-hydroxybutyrate (Fig. S6) in addition to glucose. These additional substrates were able to correct the impaired basal OCR in the cerebral cortical synaptosomes, but not in the hippocampal synaptosomes, of the 5xFAD mice. This indicates that cerebral cortical synapses maintain the metabolic capacity to utilize alternative substrates, whereas hippocampal synapses do not, implying an overall hampered synaptic mitochondrial capacity in the 5xFAD hippocampus. The machinery of mitochondrial energy production, the electron transport chain and the ATP synthase, are located within the mitochondrial cristae [64]. Cristae modulation directly affects cellular metabolism [65] and is an integral part of cellular apoptosis [64]. In line with our functional metabolic studies, we found a larger occurrence of cristaeless mitochondria in the 5xFAD hippocampus, which could indicate that mitochondrial changes not only cause bioenergetics deficits, but may also be involved in processes related to cell death in early AD [59]. Interestingly, it has previously been found that regional mitochondrial dysfunction, in the advanced stages of amyloid pathology, correlates with amyloid burden independent of the region of origin [66]. This is in sharp contrast to our findings, in which hippocampal mitochondrial function was highly impaired, when compared to the cerebral cortex, despite a much lower regional amyloid burden. This underlines that, in the early stages of amyloid pathology, not only amyloid burden, but also intrinsic regional mitochondrial properties may drive mitochondrial dysfunction.

## Materials and methods

### Materials

The stable isotopes [U-^13^C]glucose (CLM-1396, 99%), [U-^13^C]glutamate (CLM-1800-H, 98%), and [U-^13^C]D-β-hydroxybutyrate (CLM-3853, sodium salt, 97%) were all from Cambridge Isotope Laboratories (Tewksbury, USA) and [1,2-^13^C]acetate (282014, sodium salt, 99%) was from ISOTEC (St. Louis, MO, USA). Mouse anti-Aβ-antibody W0-2 (MABN10) was from Millipore (Billerica, MA, USA) and goat anti-mouse antibody (BA-9200) was from Vector Laboratories (Burlingame, CA, USA). Primers for genotyping were from Eurofins Genomics (Aarhus, Denmark). All other chemicals used were of the purest grade available from regular commercial sources. Experiments were reported according to the ARRIVE guidelines.

### Animals

Transgenic male 5xFAD mice (TG(APPSwFlLon,PSEN1*M146L*L286V)6799Vas, Jax strain: 006554) and wild-type females (Jax strain: 100012), both on B6/SJLF1J background, were from Jackson Laboratories (Bar Harbor, ME, USA) and a colony was bred and maintained at the Department of Drug Design and Pharmacology, University of Copenhagen. The 5xFAD mice express five mutations of familial AD in the amyloid precursor protein (APP) and presenillin1 (PSEN1) genes under the neuron-specific Thy1 promoter, leading to rapid brain amyloid deposition [17]. The mice were housed together in individually ventilated cages in a specific pathogen-free, humidity- and temperature-controlled facility with 12 h light/dark cycle and free access to water and chow. Male heterozygote 5xFAD mice were used at 2 and 4 months of age (8–9 and 16–18 weeks, respectively), and wild-type littermates were used as controls. Since 5xFAD mice display sex-specific variations in the development of brain amyloid pathology [17], only male mice were included in this study. Mice were genotyped from ear clippings by a standard PCR protocol (Jax protocol: 23370) for the APP gene using the following primers: transgene forward: *AGG ACT GAC CAC TCG ACC AG* (olMR3610), transgene reverse: *CGG GGG TCT AGT TCT GCA T* (olMR3611), internal positive control forward: *CTA GGC CAC AGA ATT GAA AGA TCT* (olMR7338), internal positive control reverse: *GTA GGT GGA AAT TCT AGC ATC ATC C* (olMR7339).

### Quantification of Aβ by immunohistochemistry

Quantification of cerebral amyloid-β (Aβ) load was performed as described in detail in ref. [48]. Briefly, 5xFAD and control mice were transcardially perfused with ice-cold saline and followed by 4% PFA solution. The brains were post-fixated in 4% PFA for 4 h at 4 °C and subsequently transferred to first a 30% sucrose solution and then an antifreeze solution and stored at −20 °C. Frozen tissue was cut on a microtome (35 µm) (Leica SM 2000R). Sections were preheated, rinsed, and incubated with the primary antibody (mouse anti-Aβ, 1:1000) overnight. Sections were subsequently incubated with the secondary antibody (goat anti-mouse, 1:400) for 2 h at 20 °C, before transfer to a solution containing mouse StreptAvidin (1:1000) for 2 h. Finally, sections were incubated for approximately 3 min in Ni-enhanced DAB solution. Sections were automatically scanned (Hamamatsu NanoZoomer XR) and analyzed using Adobe Photoshop. Aβ plaques were measured using the color range command. The final values were obtained by dividing the Aβ plaques area by the total area of the section.

### Proteomic analysis

5xFAD and control mice were euthanized by cervical dislocation. Cerebral cortical and hippocampal tissues were dissected on ice and immediately frozen at −80 °C. The two brain regions were lysed in 4% SDS buffer (100 mM Tris pH 8), sonicated for 15 min (Diagenode Bioruptor 300), and subsequently boiled at 95 °C for 10 min and then sonicated again. After centrifugation, the protein concentration was measured using BCA assay (ThermoFischer Scientific). The proteins were reduced and alkylated using 10 mM dithiothreitol (DTT) (Sigma Aldrich) and 55 mM iodoacetamide (IAA) (10 mM; Sigma Aldrich). Protein precipitation was done by using 4X ice-cold acetone overnight at −20 °C. The protein pellet was washed in acetone and resuspended in denaturation buffer (6 M urea, 2 M thiourea, 10 mM HEPES, pH = 8; Sigma Aldrich) and was sonicated for 15 min. Then, the proteins were digested with 1:100 LysC (Wako Chemicals, Japan) for 3 h at room temperature (RT). Urea concentration was diluted to 2 M using 50 mM ammonium bicarbonate and then digested with 1:100 Trypsin (Life Technologies) overnight at 37 °C. The peptides were acidified by 1% trifluoroacetic acid (TFA) (Merck) and transferred to StageTips with SDB-RPS (Empore, SigmaAldrich) for desalting and concentration. Peptides were eluted with 80% acetonitrile and 1% ammonia. The eluted samples were reduced by vacuum centrifugation at 45 °C and peptide concentration was determined by Nanodrop (Thermo Fisher Scientific) measurement at A280 nm. The sample concentrations were adjusted to 500 ng per injection in 5% ACN/1% TFA.

### Liquid chromatography-mass spectrometry (LC-MS/MS) analysis

The samples were analyzed with nanoflow Easy-nLC 1200 (Thermo Fisher Scientific, Denmark) connected to Q Exactive HF-X mass spectrometers (Thermo Fisher Scientific, Denmark). Peptides were separated on in-house packed column (75 μm inner diameter × 50 cm length) with 1.9 μm C18 beads (Dr. Maisch, Germany). Column temperature was kept at 60 °C. Peptide separation was achieved by 100 min gradients. Peptides were loaded and eluted with a nonlinear gradient of increasing buffer B (0.1% formic acid and 80% acetonitrile) and decreasing buffer A (0.1% formic acid) at a flow rate of 350 nL/min. Buffer B was increased slowly from 3% to 23% over 82 min and ramped to 40% over 8 min, and then to 98% over 6 min, where it was held for 4 min before being dropped down to 5% again for column re-equilibration. Mass spectrometer was operated in positive polarity mode with a capillary temperature of 275 °C. Full MS survey scan resolution was set to 60,000 with an automatic gain control target value (AGC) of 3 × 10^6^ using a scan range of 300−1650 *m*/*z* and maximum injection times (IT) of 15 ms. This was followed by a data-dependent higher-energy collisional dissociation (HCD)-based fragmentation (normalized collision energy = 28) of up to 15 most abundant precursor ions. The MS/MS scans were obtained at 15,000 resolution with AGC target of 5 × 10^4^ and IT of 25 ms. Peptide repeated sequencing was reduced by dynamically excluding previously targeted peptides for 30 s.

### MaxQuant data processing

All data files were analyzed using the MaxQuant software suite 1.5.5.1 with the Andromeda search engine [67]. MS/MS spectra were searched against an in silico tryptic digest of *Mus musculus* proteins from the UniProt sequence database. All MS/MS spectra were searched as previously described [19]. The match between runs option was enabled with a match time window of 0.7 min and an alignment time window of 20 min [68]. Relative, label-free quantification (LFQ) of proteins was done using the MaxLFQ algorithm integrated into MaxQuant. Protein identification needed minimum one unique or razor peptide per protein group and minimum ratio count was set to 2.

### MS data analysis and visualization

The quantitative protein abundance data generated in MaxQuant were transferred to the Perseus software suite 1.6.0.6, where data handling including normalization, annotation, statistics, enrichment analysis, and visualization was conducted [69]. First, hits to the reverse database, potential contaminants, and proteins only identified by site were excluded. A total of 5729 protein groups were identified in all the samples. We filtered for at least three quantified values out of four replicates in at least one group when analyzing all regions and ages together. For the two-sample *t*-tests, we analyzed each comparison separately to eliminate noise from imputation and to achieve high stringency and confidence. In both cases, missing values were replaced by imputation with values representing a normal distribution (downshift of 1.8 standard deviations and a width of 0.3 standard deviations). Differentially expressed proteins across region, age, and genotypes were identified by Multiple-sample ANOVA test at a permutation-based FDR < 0.05 with 250 randomizations. PCA was performed with standard settings. LFQ intensity correlations were done by Pearson correlations. Hierarchical clustering analysis was done using Euclidean distance on *Z*-scored data, preprocessing with *k*-means for 300 clusters. Volcano plots were generated using FDR < 0.05 and S0 > 0.1.

### Microwave fixation of brain tissue

5xFAD and control mice were euthanized by focused beam microwave irradiation to the head (Gerling Applied Engineering) to ensure termination of all metabolic activity [70]. The mice were decapitated, the cerebral cortical and hippocampal areas dissected and transferred to ice-cold 70% ethanol. The tissue was subsequently sonicated and centrifuged (4000*g* × 20 min), and the supernatant was removed and lyophilized.

### Metabolomic analysis using gas chromatography-mass spectrometry (GC-MS) analysis

Microwave fixated brain tissue was pulverized utilizing a Freezer/Mill Cryogenic Grinder and extracted in methanol/water (50%/50%, V/V). The brain homogenate was separated into polar and protein fractions, as previously described [71]. The protein fractions were hydrolyzed in 1 N HCl (2 h at 95 °C), after which methanol was added to a final concentration of 50%. The hydrolyzed samples were centrifuged, and the supernatant collected. The polar fractions and hydrolyzed pellets were dried by SpeedVac (Thermo). Samples were derivatized by sequential addition of metoxyamine (in pyridine) followed by silylation with *N*-methyl-*N*-trimethylsilylation and incubated for 60 min at 60 °C after the addition of each reagent. The gas chromatography-mass spectrometry (GC-MS) protocol was similar as previously described in ref. [72], with adjusted temperature gradient for GC: initial temperature was 130 °C (4 min) and increased at 6 °C/min to 243 °C, then ramped to 280 °C at a rate of 60 °C/min (2 min). An electron ionization (EI) energy of 70 eV was applied. Samples were scanned at a *m*/*z*: 50–800 range and full scan mode was employed for metabolomics analysis. Data were analyzed utilizing Masshunter Software (Agilent Technologies) and peaks were identified by employing a modified version of the Agilent Fiehn GC/MS metabolomics library. Briefly, the AMDIS (Automated Mass Spectral Deconvolution and Identification System) software was utilized to translate mass spectra to relative metabolite abundance and subsequently matched to the FiehnLib metabolomics library for retention time and fragmentation pattern. Samples were standardized to an internal technical control L-norvaline and thymine derived from the protein pellet. Relative values for the metabolites were normalized to the average across all samples and the average for each group visualized as a heatmap to demonstrate trends between genotype, brain region, and age.

### Determination of amino acid amounts by HPLC analysis

Extracts of microwave fixated brain tissue were reconstituted in water and analyzed by reverse-phase high-performance liquid chromatography (HPLC; Agilent Technologies, 1260 Inifinity, Agilent ZORBAX Eclipse Plus C18 column) to quantitatively determine the amounts of amino acids [22]. A pre-column derivatization with o-phthalaldehyde and fluorescent detection, λ_ex_ = 338 nm, λ_em_ = 390 nm, was performed. Gradient elution with mobile phase A (10 mM NaH_2_PO_4_, 10 mM Na_2_B_4_O_7_, 0.5 mM NaN_3_, pH 8.2) and mobile phase B (acetonitrile 45%: methanol 45%: H_2_O 10%, V:V:V) was performed. The amounts of amino acids were determined from analysis of standards containing the amino acids of interest.

### Ex-vivo electrophysiology, whole-cell patch clamp

5xFAD and control mice were euthanized by cervical dislocation. The brain was placed in ice-cold *N*-methyl-D-glucamine (NMDG)-containing oxygenated artificial cerebrospinal fluid (slicing-ACSF). The right hemisphere was separated with a sagittal cut and was horizontally sliced at the dorsal hippocampus (300 μm thickness) using a vibratome (Leica VT1200, Leica, Germany) while submerged in ice-cold, slicing-ACSF containing in mM: NMDG 93, KCl 2.5, NaH_2_PO_4_ 1.2, NaHCO_3_ 30, HEPES 20, glucose 25, Na-ascorbate 5, CaCl_2_ 0.5, and MgSO_4_ 10; oxygenated (5% CO_2_/95% O_2_). Slices were transferred to a heated chamber (35 °C) for 3 min and allowed to recover prior to recordings for 1 h at RT in holding-ACSF with constant oxygenation containing in mM: NaCl 92, KCl 2.5, NaH_2_PO_4_ 1.2, NaHCO_3_ 30, HEPES 20, glucose 25, Na-ascorbate 5, CaCl_2_ 2, and MgSO_4_ 2. All ACSF solutions were adjusted to 7.35 pH and 300 mOsm. A slice was placed in a microscope chamber (Olympus BX51WI, Germany, Olympus Europe) and perfused with an oxygenated recording-ACSF containing in mM: NaCl 124, KCl 5, NaH_2_PO_4_ 1.2, NaHCO_3_ 26, glucose 10, CaCl_2_ 2.7, MgSO_4_ 1.2. The perfusion flow rate was set to 1 mL/min using a 4 Head Perfusion pump (Ole Dick, Hvidovre, Denmark). Slices were visualized via a CCD camera system (PCO Sensicam, Till Photonics, Germany) attached to the microscope and the shutter was controlled using Live Acquisition software 2.2.0 (TILL Photonics, USA). Borosilicate patch pipettes (ID 1.1 mm, OD 1.5; Sutter Instruments, USA; 7–10 MΩ) were shaped using a horizontal puller (Sutter Instruments P-97) and filled with an internal solution containing, in mM: K-gluconate 144, MgCl_2_ 3, HEPES 10, NaGTP 0.3, and Na_2_ATP 4; EGTA 0.2; 285 mOsm). The liquid junction potential was calculated as +12 mV at 20 °C as previously described [73], and no correction was made. All recordings were performed with an Axoclamp 200B amplifier (Molecular Devices, USA) connected to an analog–digital converter (Digi-Data 1440 A; Axon Instruments, Molecular Devices). Signals were sampled at 20 kHz, filtered with a 5 kHz low-pass Bessel filter, and the amplifier output gain was set to 5. Pipette offset correction, capacitance compensation (70–80%), and series resistance compensation were adjusted before each recording. Only healthy neurons, determined as those with a stable holding current <150 pA, and access resistance maintained below 20 MΩ throughout the recordings were considered for analyses. After 5 min post whole-cell configuration to allow equilibration between intracellular content and patch solution, sEPSCs were obtained in voltage-clamp, gap-free mode, with a voltage command set to −60 mV. Recording epochs of 30 s samples were analyzed using Minianalysis 6.0.3 software (Synaptosoft Inc., USA) to measure sEPSCs amplitude and interevent intervals, which were then converted to frequency. Access resistance and membrane capacitance were obtained during a test pulse. Resting membrane potential was noted by the voltage value observed with the amplifier quickly set to *I* = 0 (no holding current). Rheobase and subthreshold membrane oscillations were found at current clamp mode by the application of a ramp current ranging from 0 to 500 pA within 1 s pulse.

### Brain slice incubations and metabolic mapping using GC-MS analysis

Incubation of acutely isolated cerebral cortical and hippocampal mouse brain slices was performed as previously described [74]. Briefly, 5xFAD and control mice were euthanized by cervical dislocation, decapitated, and the brains transferred to ice-cold artificial cerebrospinal fluid (ACSF) containing in mM: NaCl 128, NaHCO_3_ 25, D-glucose 10, KCl 3, CaCl_2_ 2, MgSO_4_ 1.2, and KH_2_PO_4_ 0.4, pH = 7.4. The cerebral cortical and hippocampal areas were dissected and sliced (350 µm) on a McIlwain tissue chopper (The Vibratome Company, O’Fallon, MO, USA). The slices were kept just below the surface of 10 mL 37 °C oxygenated (5% CO_2_/95% O_2_) ACSF and pre-incubated for 45 min. Subsequently, the media were exchanged for ACSF containing the stable isotopes: 5 mM [U-^13^C]glucose, 5 mM [1,2-^13^C]acetate, 0.5 mM [U-^13^C]glutamate, or 5 mM [U-^13^C]β-hydroxybutyrate and incubated for an additional 45 min. All conditions, except [U-^13^C]glucose, were further supplemented with 5 mM unlabeled D-glucose. Incubations were terminated by transferring slices into ice-cold 70% ethanol. The slices were sonicated and centrifuged (4000*g* × 20 min) and the supernatant was removed and lyophilized. The slice extracts were reconstituted in water, acidified, extracted twice with ethanol, and the metabolites were derivatized using *N*-tert-butyldimethylsilyl-*N*-methyltrifluoroacetamide [75]. Samples were analyzed by GC (Agilent Technologies, 7820 A, J&W GC column HP-5 MS) coupled to MS (Agilent Technologies, 5977E). The isotopic enrichment was corrected for the natural abundance of ^13^C by analyzing standards containing the unlabeled metabolites of interest. Data from the [U-^13^C]glucose, [1,2-^13^C]acetate, and [U-^13^C]β-hydroxybutyrate incubations are presented as the cycling ratio, describing the rate of ^13^C accumulation and thus reflecting the rate of TCA cycle [22] (Figs. 3, 4, and S3). Data from the [U-^13^C]glutamate incubations are presented as *M* + *X*, where *M* is the molecular ion and *X* is the number of ^13^C atoms present (direct metabolism of [U-^13^C]glutamate metabolism [76]).

### Isolation of non-synaptic mitochondria and determination of OCR

Isolation and analysis of regional non-synaptic mitochondria were performed as previously described [60]. Briefly, one 5xFAD and one control mouse were euthanized by cervical dislocation in tandem, decapitated, and the brains transferred to ice-cold ACSF. All procedures were performed on ice or at 4 °C. The cerebral cortices and hippocampi were dissected and mitochondria were isolated by a Percoll gradient as described previously [60]. Protein amounts were determined by the Bradford method. The oxygen consumption rate (OCR, pmol O_2_/min) of the isolated regional mitochondria was assessed at 37 °C using a Seahorse XFe96 analyzer (Seahorse Biosciences, MA, USA). Mitochondria were suspended in assay buffer containing in mM: mannitol 220, sucrose 70, KH_2_PO_4_ 10, MgCl_2_ 5, HEPES 2, and 0.2% bovine serum albumin (BSA) (fatty acid free), pH = 7.2. 2 µg of protein for the succinate condition and 4 µg of protein for the pyruvate/malate condition was added to each well and the plate was centrifuged (2000*g* × 20 min) at 4 °C. The mitochondria were provided with 37 °C assay buffer containing 10 mM pyruvate with 2 mM malate or 10 mM succinate with 2 µM rotenone (all final concentrations) and analyzed immediately. During the course of measurements, four compounds were injected in the following order: ADP (4 mM), oligomycin A (2.5 µg/mL), carbonyl cyanide-p-trifluoromethoxyphenylhydrazone (FCCP, 4 µM), and antimycin A (4 µM), all final concentrations. OCRs were calculated using the Wave software (Seahorse Biosciences). The non-mitochondrial OCR (OCR after antimycin A injection) was subtracted all previous measurements. The OCR is presented as pmol O_2_/min/µg protein. Basal OCR refers to the OCR before the addition of any compound. As a measure of general mitochondrial function, the respiratory control ratio (RCR) was calculated by dividing the OCR after ADP injection by the OCR after oligomycin A injection.

### Isolation of synaptosomes and determination of OCR and ECAR

Isolation and analysis of regional synaptosomes were performed as previously described [77]. Briefly, one 5xFAD and one control mouse were euthanized by cervical dislocation in tandem, decapitated, and the brains transferred to ice-cold ACSF. All procedures were performed on ice or at 4 °C. The cerebral cortices and hippocampi were dissected and mitochondria were isolated by a Percoll gradient as previously described [77]. Protein amounts were determined by the Bradford method. The oxygen rate (OCR, pmol/min) and extracellular acidification rate (ECAR, mpH/min) of the isolated regional synaptosomes were assessed at 37 °C using a Seahorse XFe96 analyzer (Seahorse Biosciences, MA, USA) with a protocol adapted from Hohnholt et al. [78]. Synaptosomes were suspended in salt solution containing in mM: NaCl 120, KCl 3.4, MgSO_4_ 2, CaCl_2_ 1.3, Na_2_SO_4_ 1.2, KH_2_PO_4_ 0.4, and 0.4% BSA (fatty acid free), pH = 7.4. Then, 8 µg of protein was added to each well (polyethyleneimine coated) and the plate was centrifuged (3400*g* × 20 min) at 4 °C. The synaptosomes were provided with 37 °C salt solution containing 5 mM glucose, 5 mM glucose supplemented with 5 mM pyruvate, or 5 mM glucose supplemented with 5 mM β-hydroxybutyrate (all final concentrations) and analyzed immediately. During the course of measurements, four compounds were injected in the following order: veratridine (5 µM), oligomycin A (6 µM), FCCP (4 µM), and antimycin A in combination with rotenone (2 + 2 µM), all final concentrations. OCRs were calculated using the Wave software (Seahorse Biosciences). The non-mitochondrial OCR (OCR after antimycin A + rotenone injection) was subtracted all previous measurements. The OCR is presented as pmol O_2_/min/µg protein and the ECAR as mpH/min/µg protein). Basal OCR and ECAR refers to measurements before the addition of any compound. Synaptosomal ECAR reaches a maximum after veratridine addition [77], hence the FCCP values are not displayed.

### Ultrastructural analyses using TEM

For transmission electron microscopy (TEM), tissue was fixated in 3% glutaraldehyde (Merck) in 0.1 M sodium phosphate buffer at 4 °C for 1 h. Tissue was post-fixated in 1% osmium tetroxide. Next, tissue was dehydrated in a graded series of ethanol, then embedded in Epon (TAAB). Semi-thin sections (2 μm) were cut using a glass knife and ultramicrotome (Leica Ultracut, Leica Microsystems, Wetzlar, Germany), and sections were stained with 1% toluidine blue (Millipore) and 1% Borax (LabChem). Ultra-thin sections (50–70 nm) were then cut using a diamond knife (Jumdi, 2 mm) and the ultramicrotome, and contrasted in 2% uranyl acetate (Polyscience) and lead citrate (Merck). The samples were examined in a Philips CM100 transmission electron microscope, and images were obtained using Olympus Morada camera and iTEM software (Olympus). Morphometry was used for quantitative evaluation of mitochondria, synapses, and synaptic vesicles. Cerebral cortical and hippocampal tissues were evaluated, and three replicates/sections were analyzed per mouse/condition. Ten random locations were selected per section, and stored by their *X* and *Y* coordinates using CompuStage, at low magnification. Images from each spot were obtained at high magnification, and analyzed in Fiji. First, the relative synapse-to-cytoplasm and synaptic vesicles per synapse area ratios were evaluated. The mitochondrial analyses consisted the mean relative individual mitochondria area, the relative mitochondria-to-cytoplasm area ratio and the relative mitochondria-to-synapse area ratio. Furthermore, mitochondrial structure was assessed by the presence or absence of cristae.

### Experimental design and statistical analyses

Graphs are presented as mean ± standard error of the mean (SEM), with individual data point shown. Sample sizes were based on the variance of pilot studies and previous experiments. No randomization was applied since two defined littermate groups (5xFAD and control) were used continuously for the experiments. Investigators were blinded during data analysis. No analyzed samples were excluded. All data points are biological replicates (i.e., from individual animals) unless otherwise noted. In most cases, two independent groups were compared (5xFAD vs. control) and Student’s unpaired *t*-test (two-tailed) was applied, corrected for multiple comparisons using the Benjamini-Hochberg procedure with a critical value for false discovery of 0.10 [79] unless otherwise noted. The electrophysiological data were first tested for normality using the D’Agostino-Pearson normality test with subsequent testing for significance with either Student’s unpaired *t*-test or Mann-Whitney test. The significance level in all cases was set at *p* < 0.05 and is indicated with a single asterisk.

## Supplementary information

Supplemental information.

## Data Availability

All data of this study are available from the corresponding authors upon request. The MS-based proteomics data from this study have been deposited at the ProteomeXchange Consortium with the accession number: PXD025240 via the Proteomics Identification Database (PRIDE) partner repository.
