# Supplementary material for: Hippocampal disruptions of synaptic and astrocyte metabolism are primary events of early amyloid pathology in the 5xFAD mouse model of Alzheimer’s disease
Source: Cell Death Dis. 2021 Oct 16;12(11):954. doi: 10.1038/s41419-021-04237-y (PMC8520528; doi:10.1038/s41419-021-04237-y)
Supplement: Supplementary file 1 — Supplemental information. [file 41419_2021_4237_MOESM1_ESM.pdf]

Supplemental Information

Hippocampal disruptions of synaptic and astrocyte metabolism are primary events of early amyloid pathology in the 5xFAD mouse model of Alzheimer's disease

Jens V. Andersen, Niels H. Skotte, Sofie K. Christensen, Filip S. Polli, Mohammad Shabani, Kia H. Markussen, Henriette Haukedal, Emil W. Westi, Marta D. Castillo, Ramon C. Sun, Kristi A. Kohlmeier, Arne Schousboe, Matthew S. Gentry, Heikki Tanila, Kristine K. Freude, Blanca I. Aldana, Matthias Mann & Helle S. Waagepetersen

Content:

Figure S1: Amyloid-β (Aβ) deposition in cerebral cortex and hippocampus of control mice.

Figure S2: Supplemental proteomics analysis.

Figure S3: Unchanged ^{13}C labeling in lactate and alanine indicates unchanged glycolytic activity in brain slices from 5xFAD mice.

Figure S4: Increased cerebral cortical, but decreased hippocampal, metabolism of ^{13}C β-hydroxybutyrate in brain slices of 5xFAD mice.

Figure S5: Sustained glutamate uptake and metabolism in brain slices of 5xFAD mice.

Figure S6: Impaired oxidative and glycolytic capacity of hippocampal synaptosomes of 5xFAD mice.

Table S1: Absolute amino acids amounts of microwave fixated cerebral cortical tissue of 5xFAD mice.

Table S2: Absolute amino acids amounts of microwave fixated hippocampal tissue of 5xFAD mice.

Table S3: Passive electrical membrane properties of cerebral cortical slices of 5xFAD mice.

Table S4: Passive electrical membrane properties of hippocampal slices (CA1 region) of 5xFAD mice.

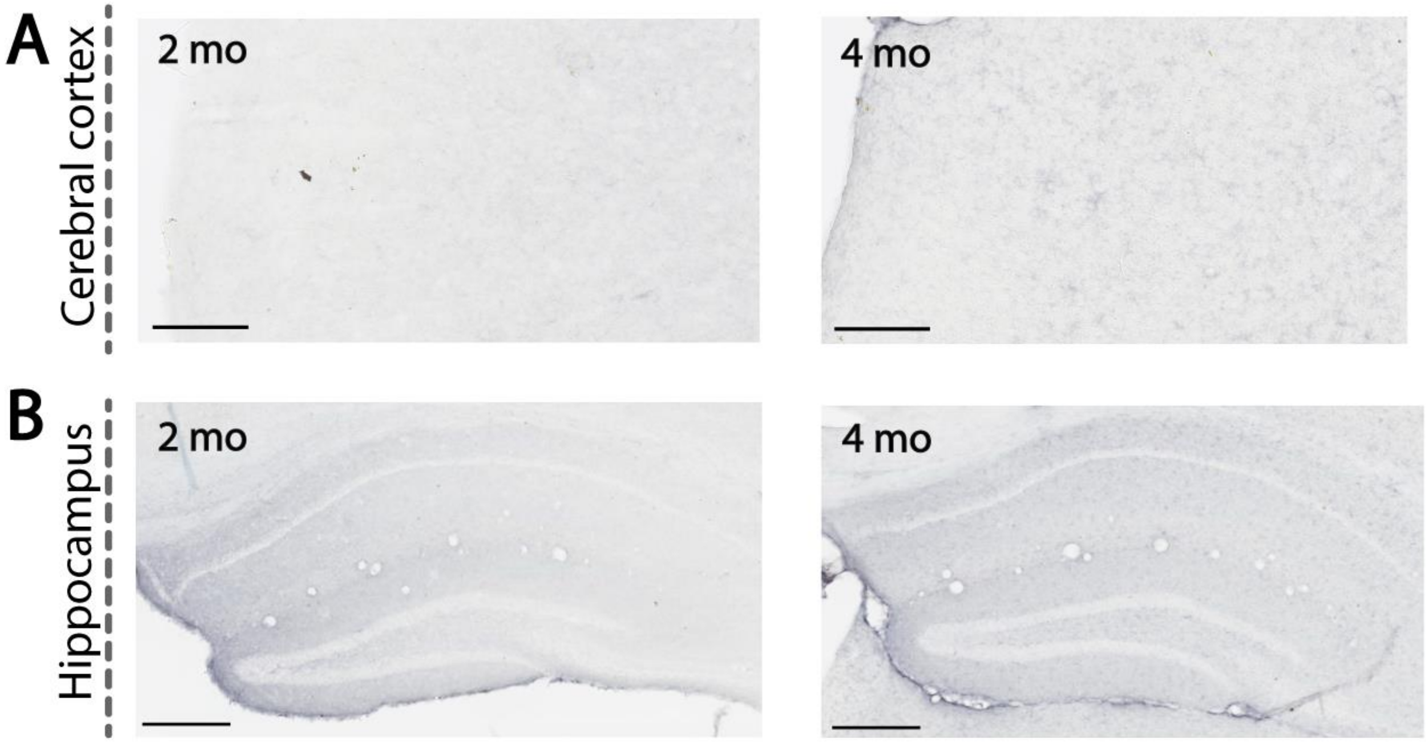

Figure S1: Amyloid-β (Aβ) deposition in cerebral cortex and hippocampus of control mice. Representative Aβ staining of the cerebral cortex (**A**) and the hippocampus (**B**) of 2 and 4 month (mo) old male control (wild-type) mice. Scale bars: A: 100 µm, B: 250 µm.

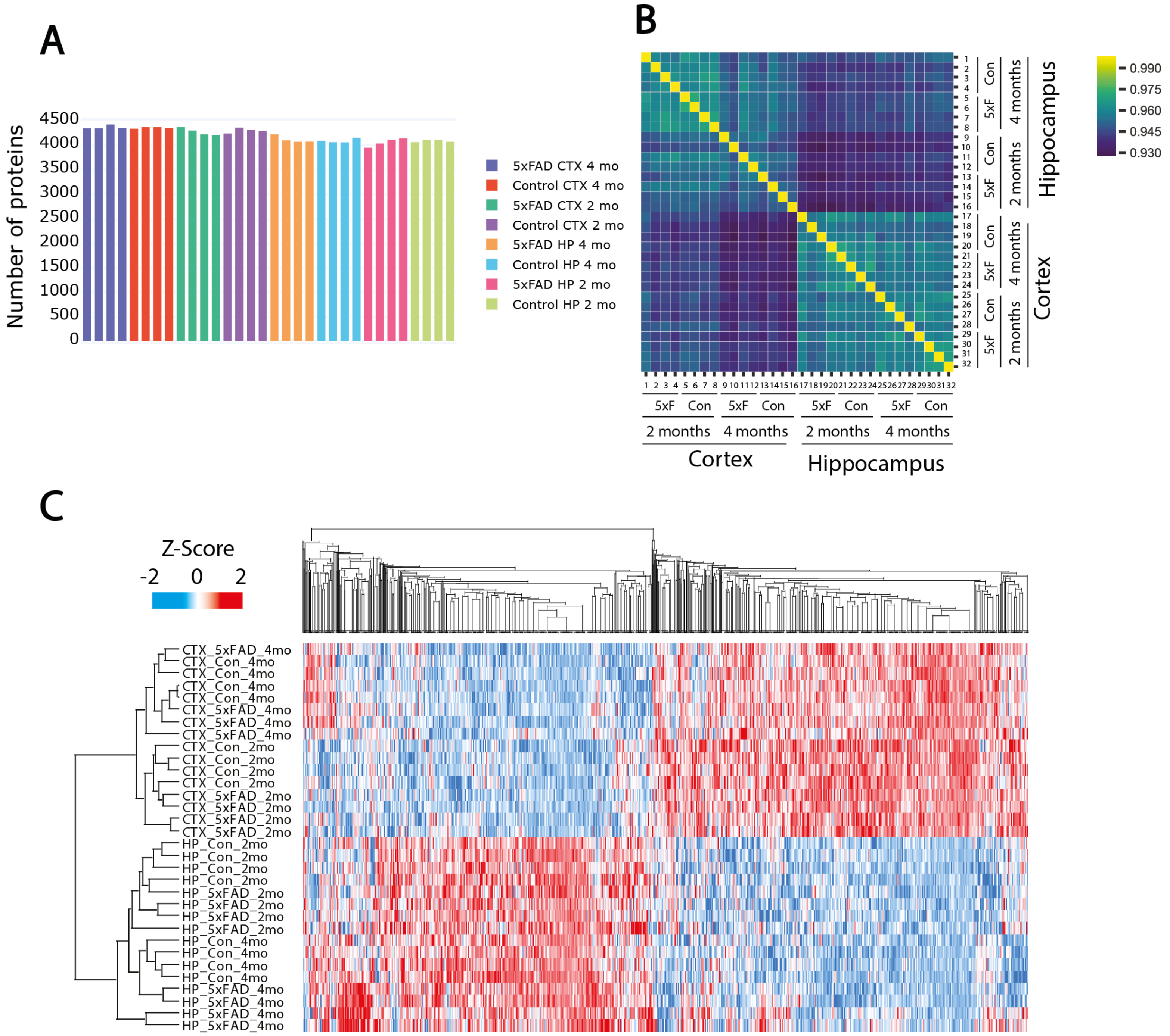

Figure S2: Supplemental proteomics analysis. (**A**) Proteins numbers after stringent filtering. (**B**) Heat-map displaying the pearson correlations between the different biological samples. (**C**) Unsupervised cluster dendrogram of the significant proteins (1997) across genotypes, age, and genotype.

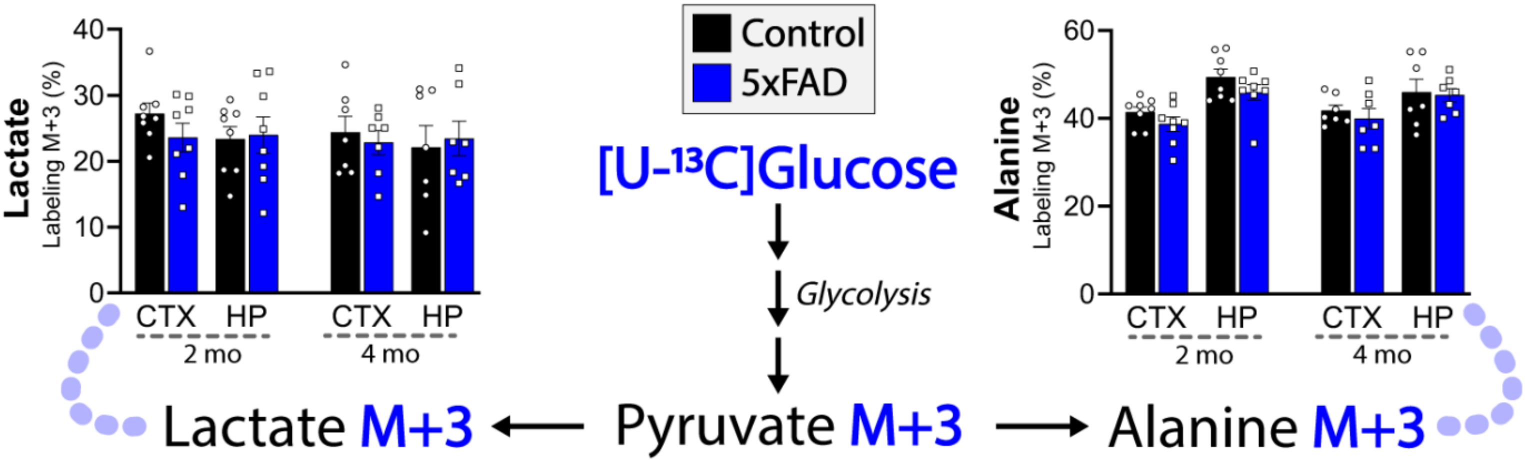

Figure S3: Unchanged ^{13}C labeling in lactate and alanine indicates unchanged glycolytic activity in brain slices from 5xFAD mice. Labeling in lactate M+3 and alanine M+3 from metabolism of [U-^{13}C]glucose in acutely isolated cerebral cortical and hippocampal brain slices of 2 and 4 months (mo) old 5xFAD mice. Lactate and alanine are generated from the end-product of glycolysis, pyruvate, and can therefore be used as indicators of glycolytic activity. CTX: cerebral cortex, HP: hippocampus. Mean ± SEM, n=6-8, Student's unpaired *t*-test with Benjamini-Hochberg correction.

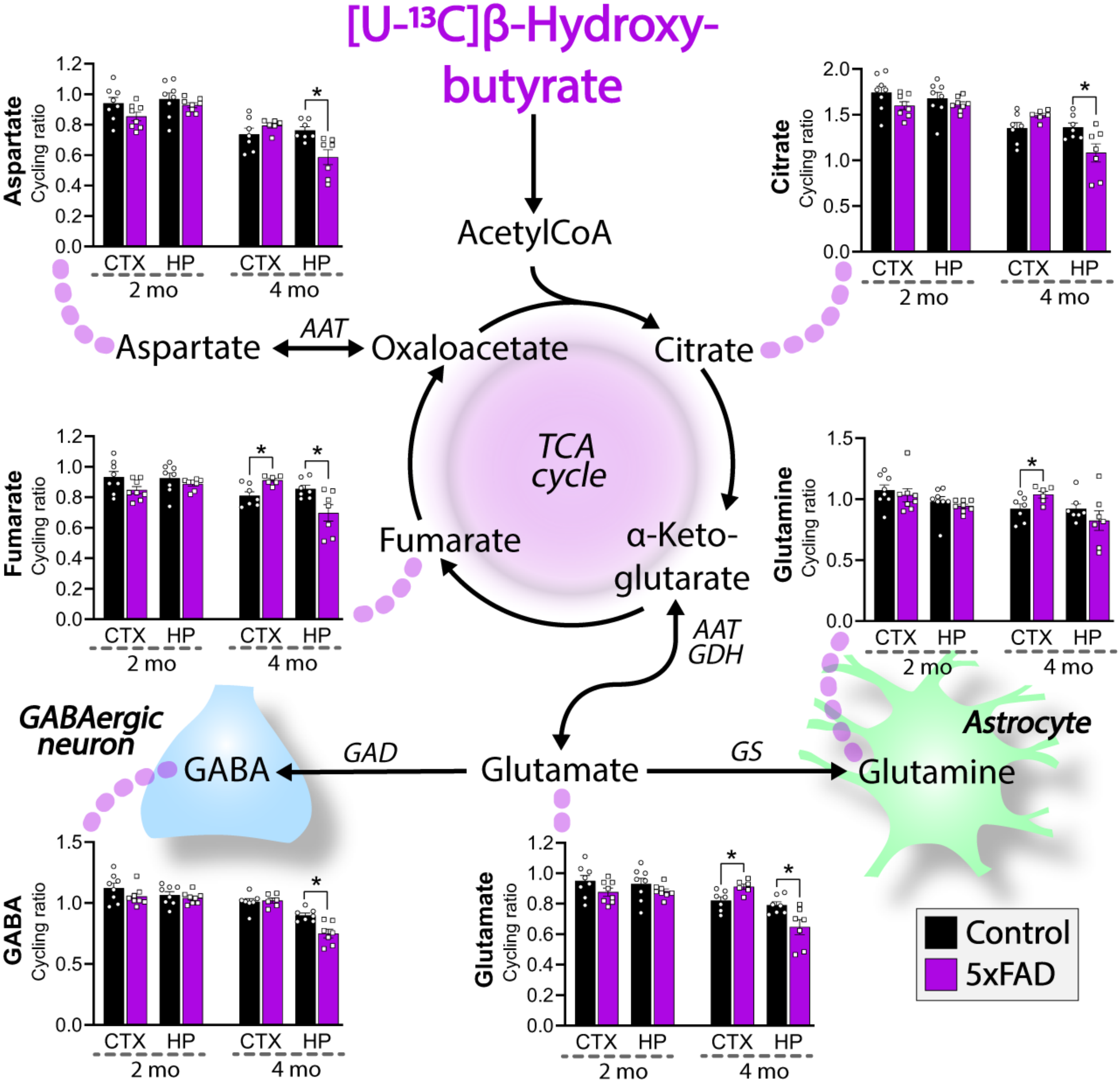

Figure S4: Increased cerebral cortical, but decreased hippocampal, metabolism of ^{13}C β-hydroxybutyrate in brain slices of 5xFAD mice. Cycling ratios, describing the rate of TCA cycling, calculated from metabolism of [U-^{13}C]β-hydroxybutyrate in acutely isolated cerebral cortical and hippocampal brain slices of 2 and 4 months (mo) old 5xFAD mice. AAT: aspartate aminotransferase, GAD: glutamate decarboxylase, GDH: glutamate dehydrogenase. CTX: cerebral cortex, HP: hippocampus. Mean ± SEM, n=6-8, Student's unpaired *t*-test with Benjamini-Hochberg correction.

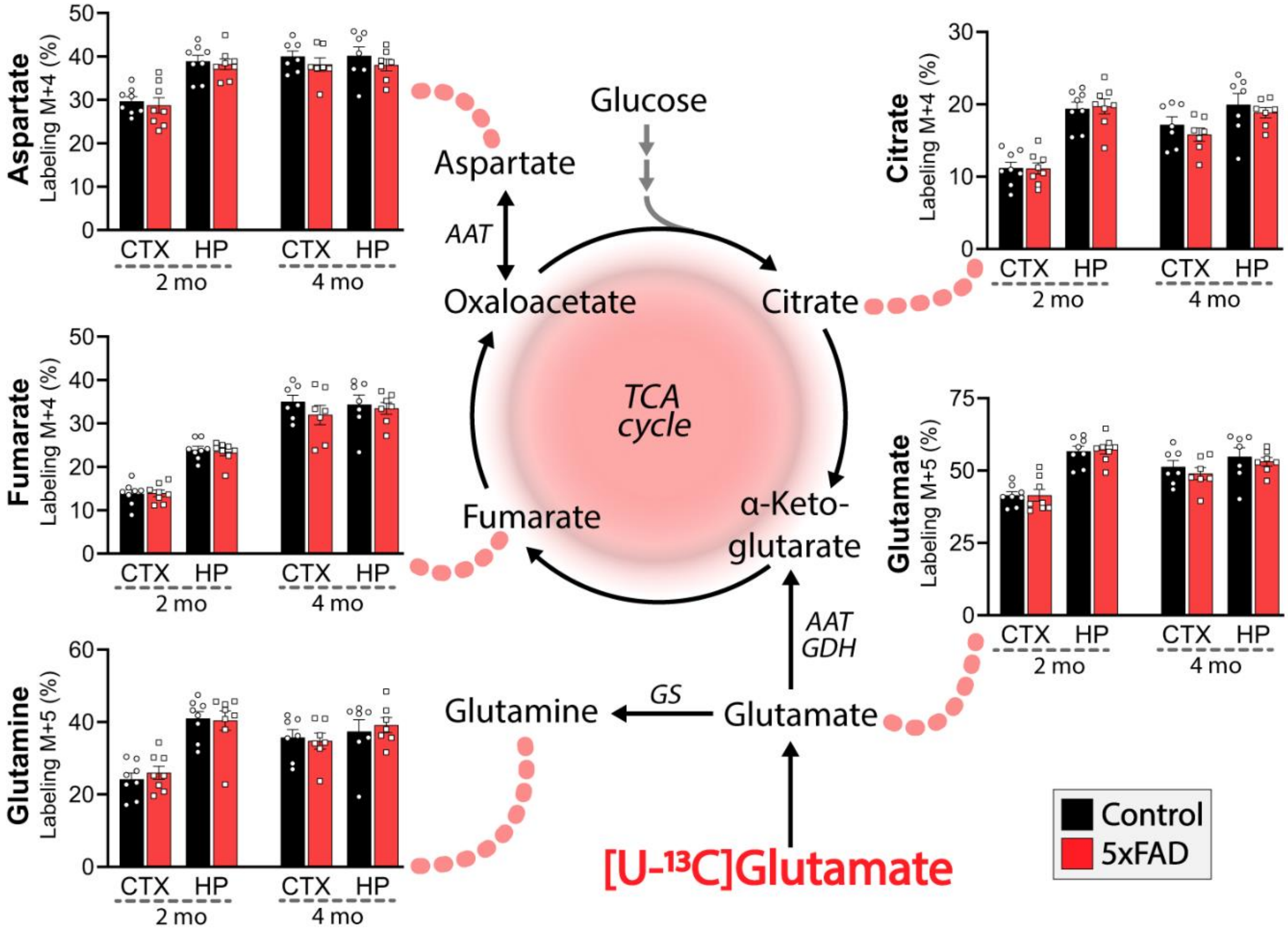

Figure S5: Sustained glutamate uptake and metabolism in brain slices of 5xFAD mice. Direct metabolism of [U-^{13}C]glutamate in acutely isolated cerebral cortical and hippocampal brain slices of 2 and 4 months (mo) old 5xFAD mice. Data is presented as the direct metabolism of glutamate as M+X, where M is the molecular ion and X is the number of ^{13}C atoms in the molecule. AAT: aspartate aminotransferase, CTX: cerebral cortex, GAD: glutamate decarboxylase, GDH: glutamate dehydrogenase, GS: glutamine synthetase, HP: hippocampus. Mean ± SEM, n=7-8, Student's unpaired *t*-test with Benjamini-Hochberg correction.

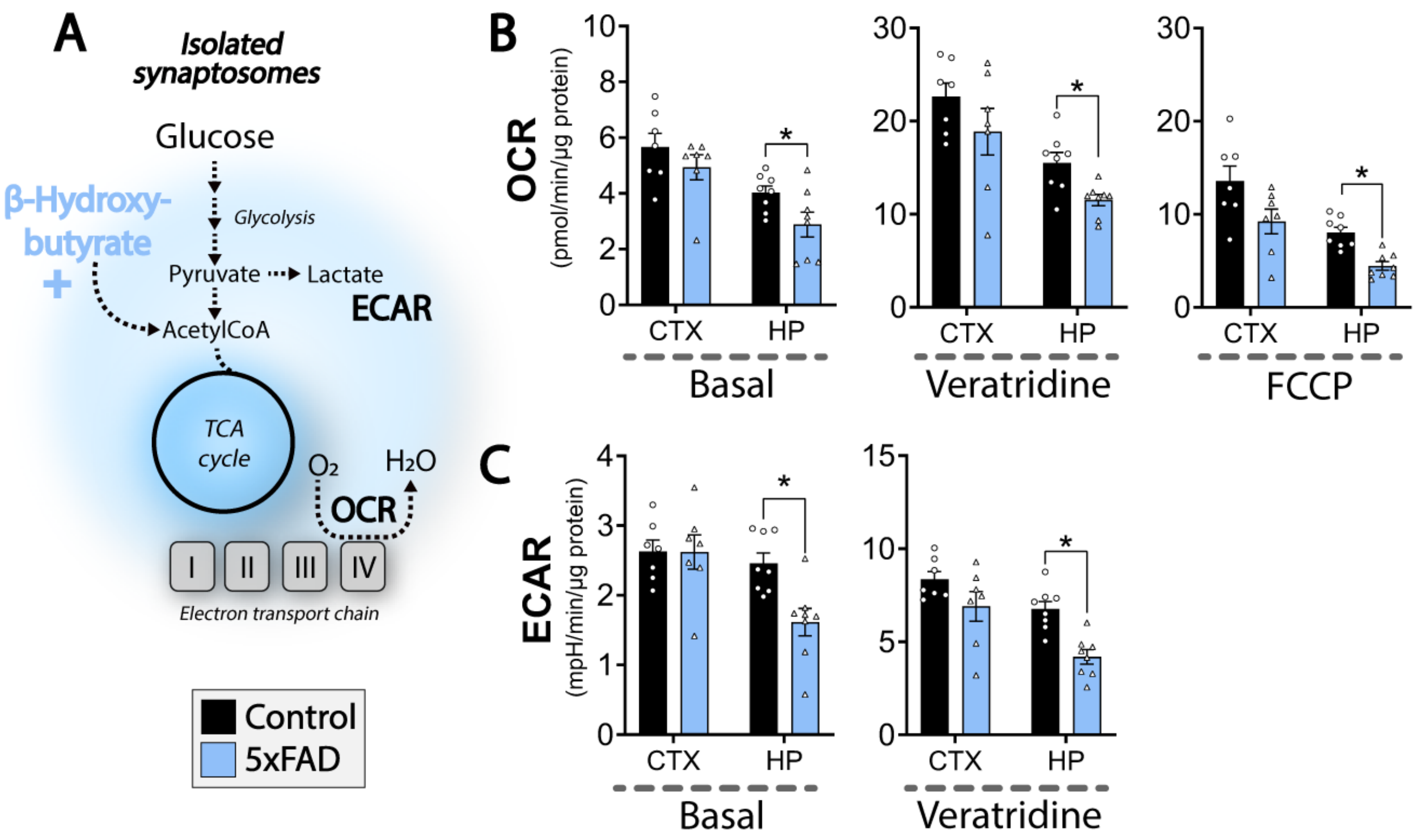

Figure S6: Impaired oxidative and glycolytic capacity of hippocampal synaptosomes of 5xFAD mice. Analysis of oxygen consumption rate (OCR) and extracellular acidification rate (ECAR) of isolated cortical and hippocampal synaptosomes of 4 months old 5xFAD mice. (**A**) The synaptosomes were provided with glucose in combination with the ketone body β-hydroxybutyrate (**B** & **C**). The synaptosomes were stimulated with veratridine, a neurotoxin inhibiting closure of voltage-gated sodium channels leading to depolarization, and with the mitochondrial uncoupler FCCP inducing maximal uncoupled respiration. Basal refers to non-stimulated OCR and ECAR. CTX: cerebral cortex, HP: hippocampus. Mean ± SEM, n=7-8, Student's unpaired *t*-test with Benjamini-Hochberg correction.

**Cerebral cortex** (nmol /mg)	2 months			4 months		
	Control	5xFAD	P-value	Control	5xFAD	P-value
Alanine	6.9 ± 0.8 (6)	4.9 ± 1.0 (6)	p = 0.16	6.8 ± 2.8 (5)	4.8 ± 0.9 (5)	p = 0.55
Aspartate	55.0 ± 7.1 (6)	45.5 ± 7.9 (6)	p = 0.39	51.8 ± 4.5 (5)	43.7 ± 5.7 (5)	p = 0.34
GABA	18.0 ± 2.2 (6)	12.9 ± 1.4 (6)	p = 0.078	22.3 ± 7.4 (5)	13.9 ± 1.1 (5)	p = 0.34
Glutamate	182.3 ± 14.4 (6)	164.0 ± 12.8 (6)	p = 0.37	231.4 ± 48.0 (5)	169.0 ± 12.3 (5)	p = 0.28
Glutamine	54.4 ± 5.2 (6)	45.0 ± 2.1 (6)	p = 0.12	56.0 ± 6.2 (5)	59.3 ± 4.3 (5)	p = 0.70
Serine	8.5 ± 0.9 (6)	7.6 ± 0.5 (6)	p = 0.37	11.2 ± 2.2 (5)	8.8 ± 0.9 (5)	p = 0.38
Taurine	95.0 ± 8.0 (6)	85.4 ± 6.9 (6)	p = 0.39	118.2 ± 15.3 (5)	97.2 ± 7.3 (5)	p = 0.29

Table S1: Absolute amino acids amounts of microwave fixated cerebral cortical tissue of 5xFAD mice. Mean ± SEM, n = 5-6 from individual animals, Student's unpaired *t*-test with Benjamini-Hochberg correction.

**Hippocampus** (nmol /mg)	2 months			4 months		
	Control	5xFAD	P-value	Control	5xFAD	P-value
Alanine	4.4 ± 0.9 (6)	4.9 ± 1.1 (6)	p = 0.73	5.8 ± 1.2 (5)	5.3 ± 1.8 (5)	p = 0.85
Aspartate	38.2 ± 7.2 (6)	32.0 ± 2.4 (6)	p = 0.43	35.8 ± 6.2 (5)	34.5 ± 4.9 (5)	p = 0.88
GABA	15.3 ± 2.4 (6)	14.2 ± 2.4 (6)	p = 0.74	17.5 ± 2.9 (5)	16.5 ± 3.9 (5)	p = 0.82
Glutamate	156.7 ± 29.5 (6)	132.2 ± 11.4 (6)	p = 0.46	144.9 ± 14.5 (5)	147.8 ± 18.4 (5)	p = 0.91
Glutamine	49.7 ± 9.8 (6)	40.6 ± 3.0 (6)	p = 0.39	40.9 ± 5.1 (5)	46.3 ± 2.8 (5)	p = 0.42
Serine	6.6 ± 1.6 (6)	6.1 ± 0.7 (6)	p = 0.78	7.4 ± 0.6 (5)	7.8 ± 1.5 (5)	p = 0.82
Taurine	86.9 ± 16.9 (6)	79.0 ± 8.8 (6)	p = 0.69	86.9 ± 4.5 (5)	89.9 ± 19.6 (5)	p = 0.82

Table S2: Absolute amino acids amounts of microwave fixated hippocampal tissue of 5xFAD mice. Mean ± SEM, n = 5-6 from individual animals, Student's unpaired *t*-test with Benjamini-Hochberg correction.

**Cerebral cortex**	2 months			4 months		
	Control	5xFAD	P-value	Control	5xFAD	P-value
Rheobase (pA)	88.2 ± 10.4 (13)	96.9 ± 8.9 (12)	p = 0.5	500.5± 80.7 (17)	356 ± 125.7 (3)	p = 0.48
Access resistance (MΩ)	9.9 ± 0.7 (22)	12.3 ± 1.2 (19)	p = 0.09	8.22 ± 1.0 (27)	10.01 ± 0.9 (16)	p = 0.24
Membrane capacitance (pF)	35.9 ± 2.1 (22)	41.4 ± 2.0 (19)	p = 0.06	38.5 ± 1.5 (22)	38.08 ± 3.4 (16)	p = 0.88
Resting membrane potential (mV)	- 62.2 ± 1.7 (19)	- 62.4 ± 2.6 (19)	p = 0.9	- 66.6 ± 1.6 (27)	- 66.02 ± 2.1 (16)	p = 0.09
Subthreshold membrane oscillations (pA)	936.9 ± 83.8 (13)	979.3 ± 88.2 (12)	p = 0.7	1369.0 ± 163.7 (17)	1333.0 ± 333.5 (13)	p = 0.93

Table S3: Passive electrical membrane properties of cerebral cortical slices of 5xFAD mice.
Mean ± SEM, N=3-27 from 4-6 pairs of animals, Student's unpaired *t*-test or Mann-Whitney test.

**Hippocampus**	2 months			4 months		
	Control	5xFAD	P-value	Control	5xFAD	P-value
Rheobase (pA)	86.2 ± 10.8 (11)	92.5 ± 8.8 (11)	p = 0.6	254.8± 24.6 (17)	234.2 ± 29.6 (13)	p = 0.59
Access resistance (MΩ)	11.0 ± 0.5 (18)	13.2 ± 0.9 (17)	p = 0.052	9.4 ± 1.1 (12)	10.1 ± 1.0 (22)	p = 0.61
Membrane capacitance (pF)	39.8 ± 3.6 (18)	42.2 ± 3 (17)	p = 0.6	44.7 ± 2.4 (21)	41.2 ± 3.3 (22)	p = 0.39
Resting membrane potential (mV)	-59.1 ±1.4 (18)	-60.8 ± 1.8 (16)	p = 0.4	-59.4 ± 1.8 (21)	-58.2 ± 1.4 (22)	p = 0.62
Subthreshold membrane oscillations (pA)	809.2 ± 49.0 (11)	841.8 ± 88.9 (11)	p = 0.7	1168.0 ± 97.5 (17)	1031.0 ± 151.0 (13)	p = 0.43

Table S4: Passive electrical membrane properties of hippocampal slices (CA1 region) of 5xFAD mice. Mean ± SEM, N=11-22 from 4-6 pairs of animals, Student's unpaired *t*-test or Mann-Whitney test.
